# A Comparative Study Based on Petrophysical and Cluster Analysis Approach for Identification of Rock Types in Heterogeneous Sandstone Reservoirs

**DOI:** 10.1021/acsomega.3c08403

**Published:** 2024-07-25

**Authors:** Muhammad Nofal Munir, Mohammad Zafar, Abid Ali, Muhsan Ehsan, Kamal Abdelrahman, Ahmed E. Radwan, Hezam Al-Awah

**Affiliations:** † Department of Earth and Environmental Sciences, Bahria School of Engineering and Applied Sciences, Bahria University, Islamabad 44000, Pakistan; ‡ Research and Development GVERSE Geographix, LMK Resources, Islamabad 44000, Pakistan; § Institute of Geology, University of the Punjab, Lahore 54590, Pakistan; ∥ Department of Geology and Geophysics, College of Science, King Saud University, P.O. Box 2455, Riyadh 11451, Saudi Arabia; ⊥ Faculty of Geography and Geology, Institute of Geological Sciences, Jagiellonian University, Gronostajowa 3a; 30-387, Kraków 31-007, Poland; # Geology Program, Department of Chemistry and Earth Sciences, College of Arts and Sciences, Qatar University, Doha 2713, Qatar

## Abstract

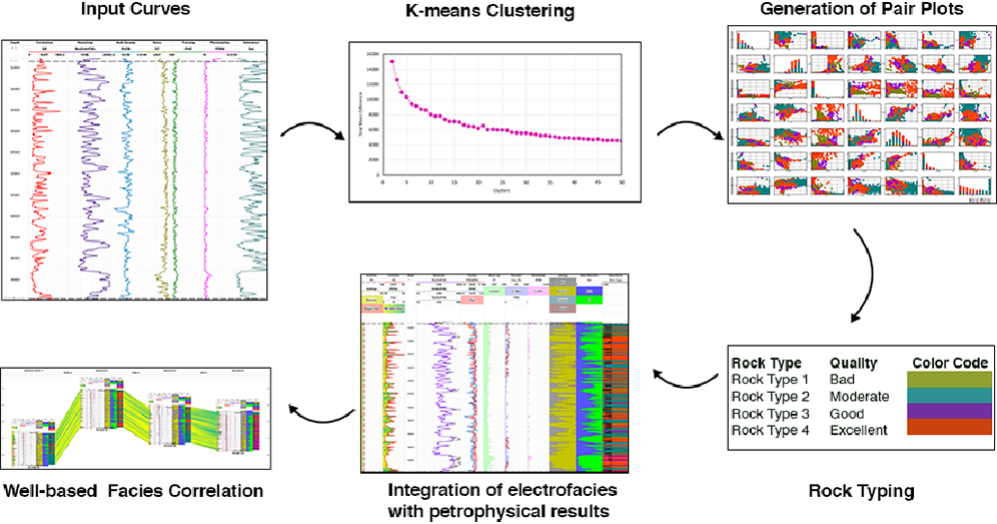

To delineate a powerful reservoir model, rock type identification is an essential task. Recognizing intervals with promising reservoir quality in a heterogeneous reservoir, such as the Pab Formation, using well logs is critical for better exploration, because coring programs are always impractical due to time and cost constraints. Rock types are described by specific log responses, which are ultimately distinguished with the help of electrofacies. The current study uses a cluster analysis technique for the evaluation of reservoir rock types in the identified sand units. K-means cluster analysis is employed to define electrofacies, which are ultimately classified into four rock types on the basis of reservoir quality, from bad to excellent. Rock typing using cluster analysis has been done for four wells, and a correlation has been made to depict changes in electrofacies. From well-to-well correlation, it can be inferred that the reservoir quality of the Pab Formation at the lower portion of Zamzama-02 and 05 wells is excellent and is defined by rock type 4. The Zamzama-03 well in the southwestern region, on the other hand, has good to moderate reservoir quality, as demonstrated by dominating rock types 3 and 2, respectively. The applied prediction technique to the studied field provides continuous rock type identification for the entire reservoir. Using this methodology in defining rock type is cost-effective, requires less time in the demarcation of zones of interest, and is more accurate than manual analysis of the heterogeneous and thick Pab Formation. The studied approach is not only useful in the exploitation of the heterogeneous Pab Formation but also can be applied to other heterogeneous sandstone reservoirs elsewhere.

## 1 Introduction

Machine learning nowadays has immense significance in geosciences, as it offers great potential to solve interpretation-related problems. The implication of machine learning methods in rock type identification can help in distinguishing pay zones from unproductive intervals.^1,2^ It leads to the construction of more reliable static reservoir models in simulation plans, as core samples are not always feasible to be considered because of time-consuming and expensive coring programs.^3−5^ Reservoir heterogeneity is connected to diversity in sedimentary rock types and their composition (e.g., lithology, cementation, texture, thickness, and grain size).^6,7^ Lithofacies are depictions of a rock in relation to its environment of deposition and the provenance of accumulated sediments.^8^ Lithofacies determination can be carried out directly from the field observation of rocks under investigation or from the interpretation of well logs. Identification of lithofacies from log responses needs standardization from core plugs, cuttings, or outcrop samples.^9,10^ In the multivariate space of logs, the objective of cluster analysis is the detection of semblances and variations among data points, which is intended for grouping them into modules, also known as electrofacies.

The prediction of rock type using machine learning and clustering techniques was applied to reservoirs in the United States and the Middle East and obtained good results.^11−13^ The classification of rock type in the Mishrif Formation in Iraq was based on input curves for gamma ray, porosity, and water saturation to identify clusters with similar characteristics.^12^ Previously, the rock types in the study area were defined with the help of cluster analysis using different input curves (Table 1, (14)). According to Munir et al.,^15^ core-calibrated porosity provides more accurate results for different reservoir parameters compared to porosities derived from conventional petrophysics.^15^ To get better prediction accuracy and reach better prediction performance for the studied heterogeneous reservoir, we have used different curves, and core calibrated effective porosity and water saturation are used, along with other curves. In the present study, rock typing of the Pab Formation is based on input curves, (i.e., raw log curves of bulk density and sonic transit time, calculated curves including core-calibrated porosity, permeability, and water saturation) (Table 1). The input parameters such as effective porosity, permeability, and water saturation are core-calibrated, and the porosity and permeability core data corroborate the accuracy of the recognized rock types. First, the technique is applied to single borehole data belonging to the Zamzama-02 well, and then the procedure is extended to a multiwell logging data set to reconstruct the multidimensional spatial distribution of clusters revealed by the well-based correlation technique. To cover different data ranges, 20 clusters were used, which were grouped into a manageable number of rock types by classifying them into four homogeneous groups. The identified rock types are validated by petrophysical results, including lithology derived from specialized user-defined equations and other reservoir parameters of the Pab Formation.

**Table 1:** Comparison of Input Curves Used in Previous Study and Current Study

sr no.	input curves used in previous study	input curves used in current study
1	gamma ray (GR)	gamma ray (GR)
2	effective porosity (PHIE)	resistivity (ResD)
3	saturation of water (Sw)	bulk density (RHOB)
4	permeability (k)	sonic transit time (DT)
5		core calibrated PHIE
6		core calibrated Sw
7		permeability (k)

K-means cluster analysis is executed for the rock type identification of the Pab Formation. K-means clustering is a machine learning algorithm that is used for geological and geophysical analyses in the oil and gas industry.^16^ It is an unsupervised machine learning algorithm, i.e., it does not require training on already labeled data. Instead, it attempts to group observations into k clusters, where each observation is assigned to the cluster with the nearest mean or cluster centroid.^17^ The facies variable here refers to the lithofacies, which is generally defined for a sedimentary rock based on observations of grain size and mineralogy.^18^ A given facies is generally associated with a particular environment of deposition.^19,20^ Among the steps for well planning decisions, reservoir characterization is the essential one, and estimation of physical parameters, including porosity and permeability, is the basic requirement in the characterization workflow.^21^ There is more preservation of oil and gas in the voids of rocks with greater porosity, whereas permeability defines the capacity of rocks to transfer fluids.^22^ These two types of reservoir parameters are determining factors for reserve estimation and oil or gas production.^23^

Based on similarities and dissimilarities among groups, the purpose of cluster analysis is to categorize log records into groups that are outlying externally and analogous internally. The machine learning algorithm K-means clustering is being used for the demarcation of zones having excellent reservoir quality in heterogeneous sandstones of the Pab Formation.^24^ The current study uses a cluster analysis technique for the evaluation of reservoir rock types in the identified sand masses. Rocks’ physical properties and hydrocarbons in the volume under investigation by a logging tool are depicted by a distinctive set of logs termed electrofacies.^25^ The rock type shows zones of reservoirs having a homogeneous relationship among effective porosity, permeability, and water saturation. First, clustering is performed on the basis of log curves in order to find similar patterns at various depths in different wells. Later, a comparison is made between clusters generated by the K-means algorithm and lithology and reservoir parameters interpreted at the same depths to see the similarities and differences. Rock typing using cluster analysis has been done for four wells, and a correlation has been made to depict changes in electrofacies.

When clustering all petrophysical variables in a joint procedure, the lithological properties, the fluid, and other reservoir characteristics are considered to differentiate the pay zones from unproductive intervals. Since the resulting prediction is continuous along the drill core, the use of this methodology in defining rock types is cost-effective, less time-consuming in the demarcation of zones of interest, and more accurate than manual analysis of the heterogeneous and thick Pab Formation. This approach can be used as a powerful visualization tool for uncored but logged wells.

## 2 Methodology

**Figure 1:**
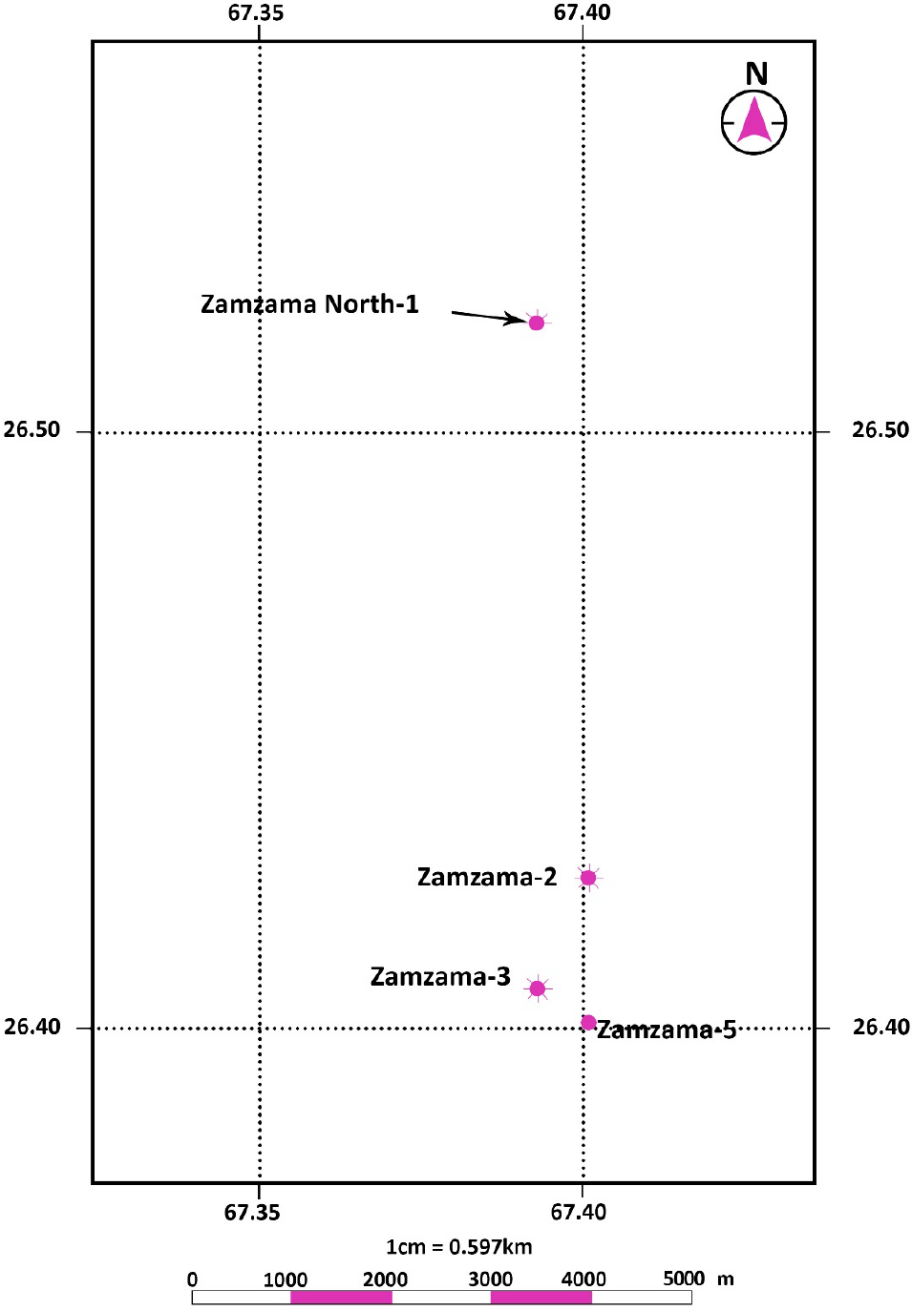
Base map showing location of Zamzama North-01, Zamzama-02, Zamzama-03, and Zamzama-05.

**Figure 2:**
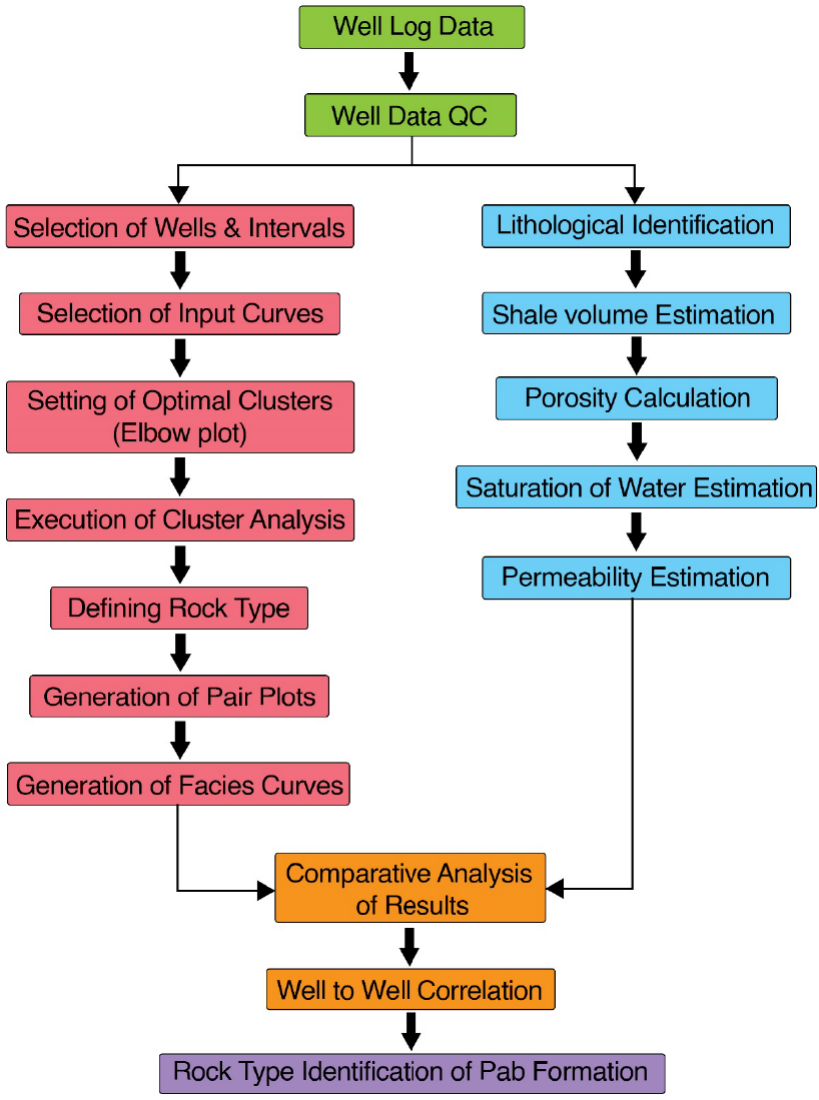
Workflow of the methodology adopted in this study.

Currently, the primary approach to discerning a specific lithology involves creating a cross-plot of conventional well logging responses.^45−47^ The method applied in this study incorporates good data for cluster and good log analysis. The study was focused on rock type identification of the Pab Formation in four wells of the Zamzama Gas Field (Figure 1). The K-means clustering was executed on the petrophysics module of GVERSE GeoGraphix, a powerful mapping and prospect interpretation tool. Complete quality assurance of well data has been performed in order to make sure that input curves have no null or exaggerated values.^26^ A workflow was established for the accomplishment of petrophysical and cluster analyses in the study area. The workflow is presented in Figure 2. The input curve data used for cluster analysis are gamma ray (GR), resistivity (ResD), bulk density (RHOB), sonic transit time (DT), effective porosity (PHIE), water saturation (Sw), and permeability (k). In order to take account of the variation in the entire data, 20 clusters have been used. Initially, the assumed mean value from input data was assigned to each cluster using the k-means technique, and then between the cluster mean value and data points, the sum of squares difference within the cluster was minimized.^18^

Cluster analysis is executed using different methods, which can be categorized into two approaches: the hierarchical approach and the nonhierarchical approach. The hierarchical approach comprises different methods, including the nearest neighbor method, where the distance between two clusters is the smallest distance between members; the average distance method, where the distance between two clusters is the average distance between members; and the furthest neighbor method, where the distance between two clusters is the maximum distance between members. The Euclidean distance relation is employed in order to measure the distance between two subjects; its mathematical relation is given as follows:^27^
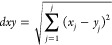


In the current research, a nonhierarchical approach for cluster analysis was employed. This approach was used when large data sets were involved, as it permitted the iterative movement of subjects between different clusters.^28,29^

### 2.1 K-means Clustering Method

In this nonhierarchical approach, suitable cluster numbers are specified, from which the ideal cluster number is selected. The clustering process is composed of two main stages. In the first stage, in order to incorporate the entire variety of detected log data ranges, log data were classified into controllable cluster numbers. For most of the data sets, a 15–20 cluster range was considered appropriate. In the second stage, clusters are grouped into adaptable rock types for reduction of the data into 4–5 identical groups.^30^

Execution of cluster analysis starts with the selection of wells and data intervals for the analysis; here, investigated wells Zamzama-02, 03, 05, and Zamzama-North-1 are selected. The data interval for the Pab Formation was selected for all of the above-mentioned wells. Before the selection of log curves as input data, data preprocessing was carried out to remove invalid log values. Over- and underestimated values were normalized using the digital curve normalization tool in GVERSE Petrophysics. The next step is to select the curves; seven input curves were included in the cluster analysis. Raw log curves include gamma, resistivity, sonic, and bulk density, whereas computed curves include core-calibrated effective porosity, permeability, and water saturation. Log curves were selected based on their measuring capabilities, like the GR and RHOB logs, which measure the rock type based on lithology, and resistivity log reflects hydrocarbon saturation. Porosity and permeability measure the reservoir’s storage and deliverability capacity, whereas saturation of water gives an indication of the presence of hydrocarbons in the reservoir of interest.^31^

**Figure 3:**
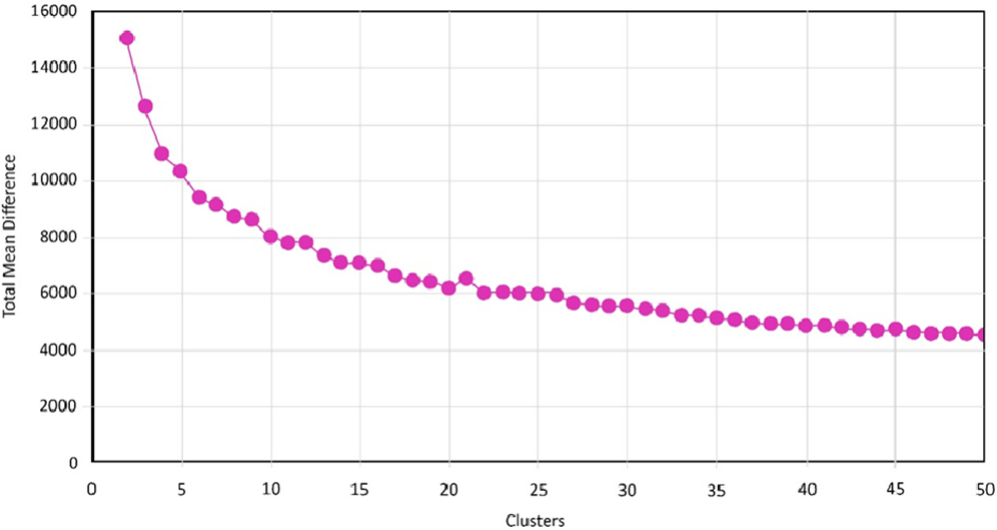
Elbow plot showing the information about cluster number and the total mean difference.

The next step involves defining the number of clusters for analysis, which is 20. An Elbow plot is generated to visually analyze the trend of clusters and their effectiveness in using that number of clusters.^32^ The Elbow method aids in determining the optimum number of clusters for the analysis. Different numbers of clusters were given to see the calculated distortion for each number of clusters. Looking for an “elbow” in the plot corresponds to the optimum number of clusters. The correct K value was found to be 20, as at this turning point, it was similar to Elbow’s method (Figure 3). The clusters are shown along the *x*-axis, while the total mean difference of the clusters is shown along the *y*-axis.

This chart plots the sum of square distances of each point from its assigned cluster for a range of values of “k”. The “elbow” is the point beyond which diminishing returns might not justify the cost of using more clusters.

After the number of clusters was defined, the execution of cluster analysis was carried out, which allowed one to witness the results of cluster analysis in a grid where any cluster or its properties could be fine-tuned. Here, logs with homogeneous values were grouped into four rock types as shown in Table 2.

**Table 2:**
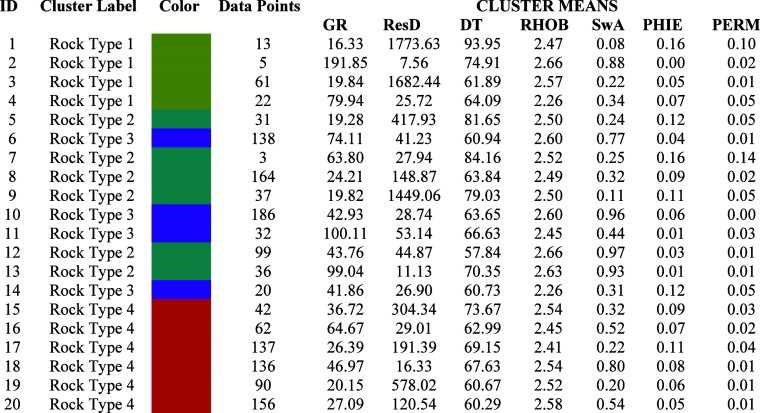
Showing Mean Value of Each Input Curve in a Grid

A pair plot was generated in order to visualize the distribution of each feature for wells and also cross-plot the features against one another. This pair plot method helps in cross-plotting quantitative data for selected pairs of variables in a data frame. These plots show that some rock types show dominant facies. Clustering has been successful at identifying consistent groupings of the well log data, both within and between the wells.

In the last step of cluster analysis, an output curve named “Rock Type” was generated, which is the output of the electrofacies log for clusters identified by the K-means algorithm and saved with input wells. After the generation of the output curve for cluster analysis, it is displayed along with the input curves in the form of colors and lines on a specialized electro facies track.

## 3 Results and Discussions

Rock type identification is carried out on the basis of a cluster analysis technique. Identification of rock type for the Late Cretaceous Pab Formation in the Zamzama Gas Field using cluster analysis is carried out on the GVERSE GeoGraphix 2022.1 software suite GVERSE Petrophysics. In drilled wells with applicable well log data, the detection of logacies is a recurrent approach. The K-means cluster analysis technique can be used in the classification of well log data into discrete classes. Rock typing was done based on electrofacies analysis using K-means cluster analysis. The results of K-means cluster analysis were validated by conventional petrophysical analysis results such as lithology, porosity, permeability, and saturation of water. The best-suited results of petrophysical parameters were positively correlated to rock type. The entire section of the Pab Formation is categorized into rock types from bad quality to excellent quality. Rock typing is carried out in such a way that it separates the sandstone of the Pab Formation from the shaly sands. In the sandstone zones of the formation, the values of porosity, permeability, and water saturation show good values, and such zones were defined as rock types having excellent reservoir quality. Shale volume was lower in rock types that have excellent quality.

### 3.1 Reservoir Characterization and Rock Typing

The characterization and classification of reservoir rock types are considered essential components of reservoir studies. The primary goal of this analysis was to identify the major rock types that are present in reservoirs. The building blocks of geological models are different rock types, because they each have a distinct reservoir property and a similar depositional and diagenetic history.^33^ The manual methods of rock type identification in heterogeneous reservoirs were inaccurate and took considerable time.^24,34^ Here, we characterized and identified the rocks types of the Pab Formation using an integrated approach. In this approach, electrofacies analysis is carried out by using the K-means clustering technique in order to separate rock types of different quality. The volume of shale estimation in heterogeneous sandstone like the Pab Formation gives an overestimation as shale does not follow the linear relationship between gamma ray index and volume of shale.^35^ Nonlinear methods and their respective corrections are applied in the present study to best estimate shale volume.^36^ In the second step, the results of the cluster analysis are validated based on lithology identified through 3 and 4 mineral modeling and other reservoir parameters. Lastly, well-to-well correlation on the basis of this interpretation is carried out in order to find how rock type is segregated between different wells in the Zamzama Gas Field.

**Figure 4:**
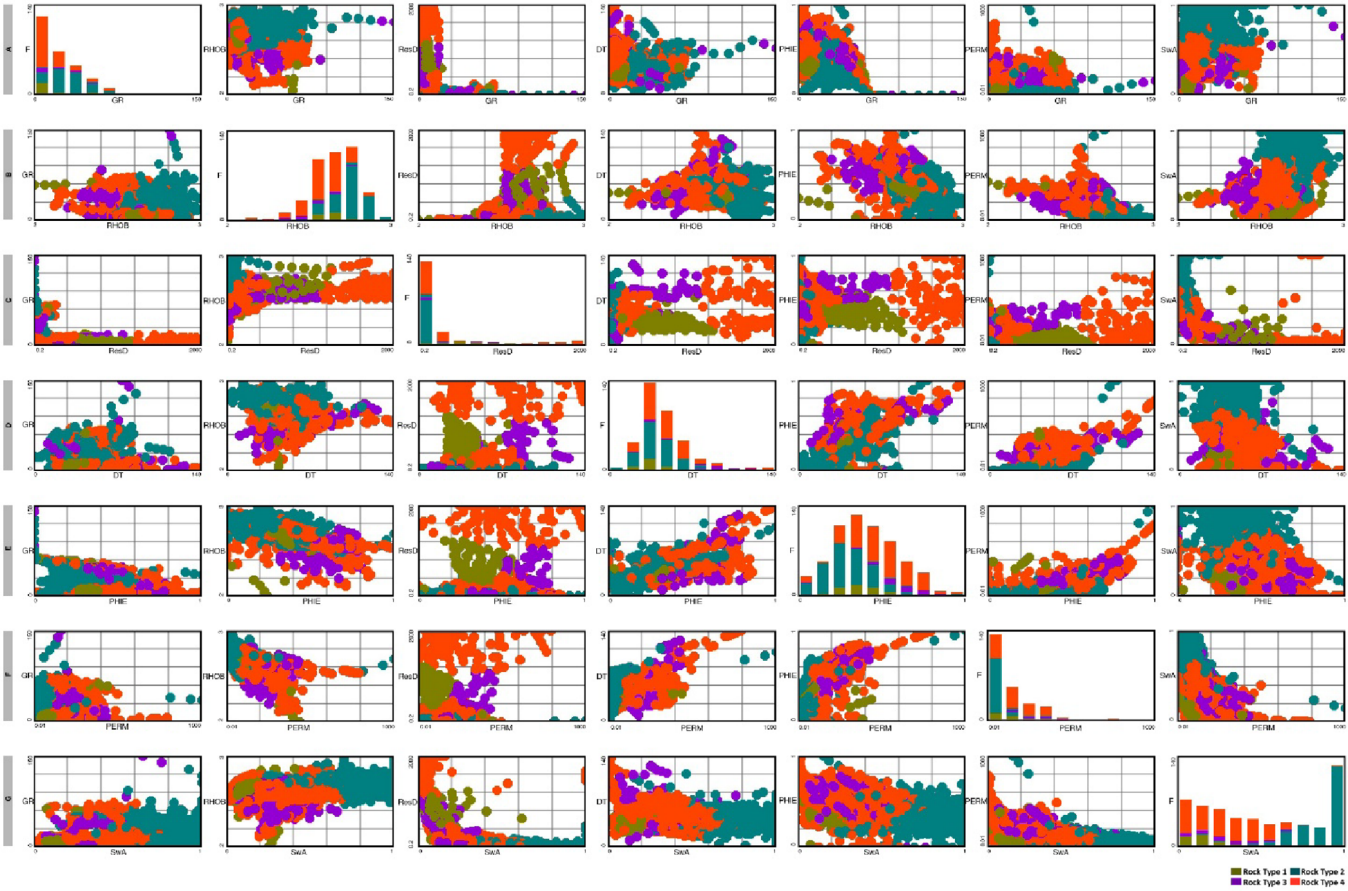
Pair plot showing crossplots among GR, RHOB, ResD, DT, PHIE, PERM, and SwA to visualize distribution of each feature for Pab Formation data interval. (A) Data plotted between GR on the *x*-axis and frequency, RHOB, ResD, DT, PHIE, PERM, and SwA on the *y*-axis. (B) Data plotted between RHOB on the *x*-axis and GR, frequency, ResD, DT, PHIE, PERM, and SwA on the *y*-axis. (C) Data plotted between ResD on the *x*-axis and GR, RHOB, ResD, frequency, DT, frequency, PERM, and SwA on the *y*-axis. (D) Data plotted between DT on the *x*-axis and GR, RHOB, ResD, frequency, PHIE, PERM, and SwA on the *y*-axis. (E) Data plotted between PHIE on the *x*-axis and GR, RHOB, ResD, DT, frequency, PERM, and SwA on the *y*-axis. (F) Data plotted between PERM on the *x*-axis and GR, RHOB, ResD, DT, PHIE, frequency, and SwA on the *y*-axis. (G) Data plotted between SwA on the *x*-axis and GR, RHOB, ResD, DT, PERM, and SwA on the *y*-axis.

**Figure 5:**
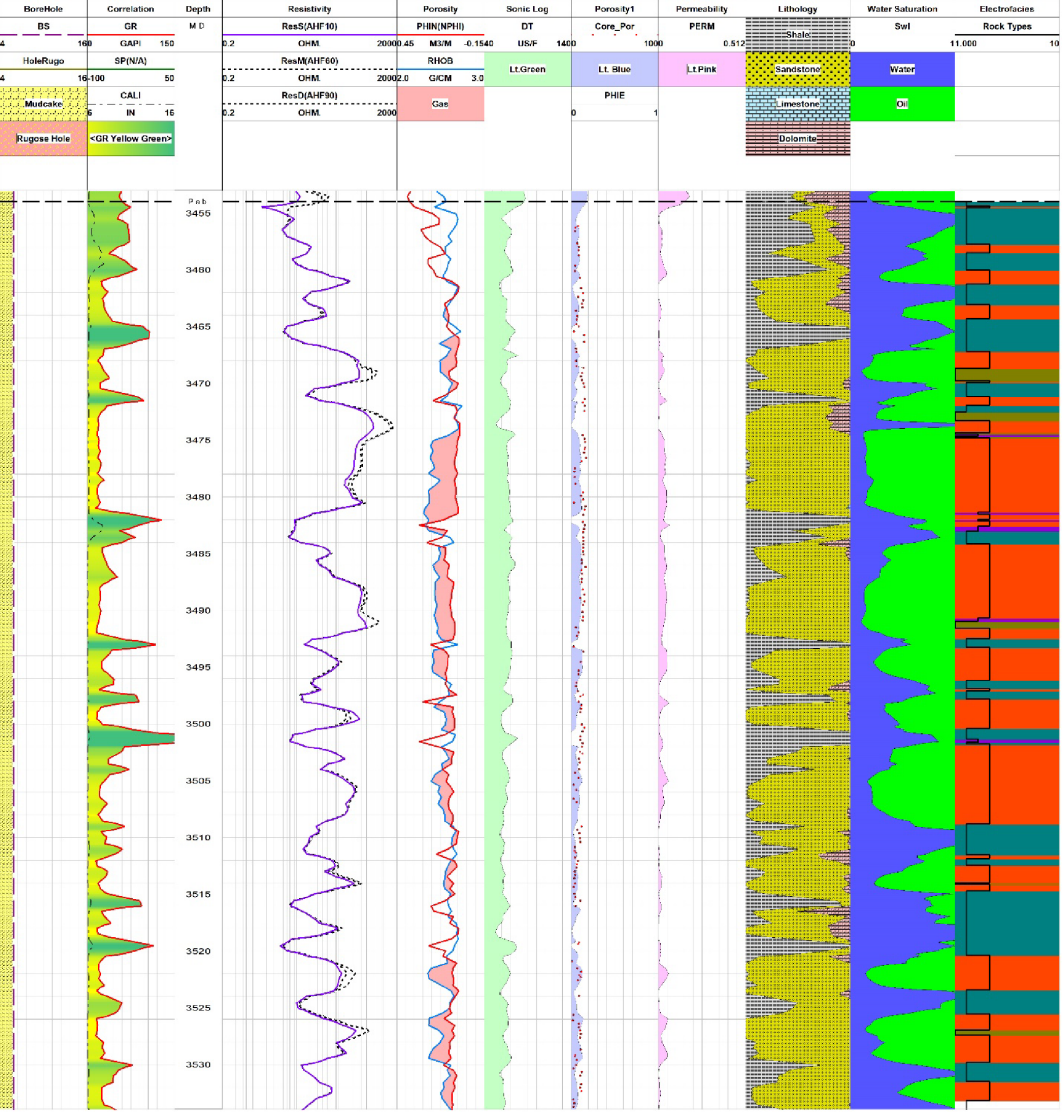
Pab Formation section in Zamzama-02 well showing results of petrophysical analysis along with rock type in the electrofacies track depicting rock reservoir quality. Reservoir quality is displayed by a curve named as Rock Types in the electrofacies track along with different colors illustrating individual rock types as shown in Table 3.

**Figure 6:**
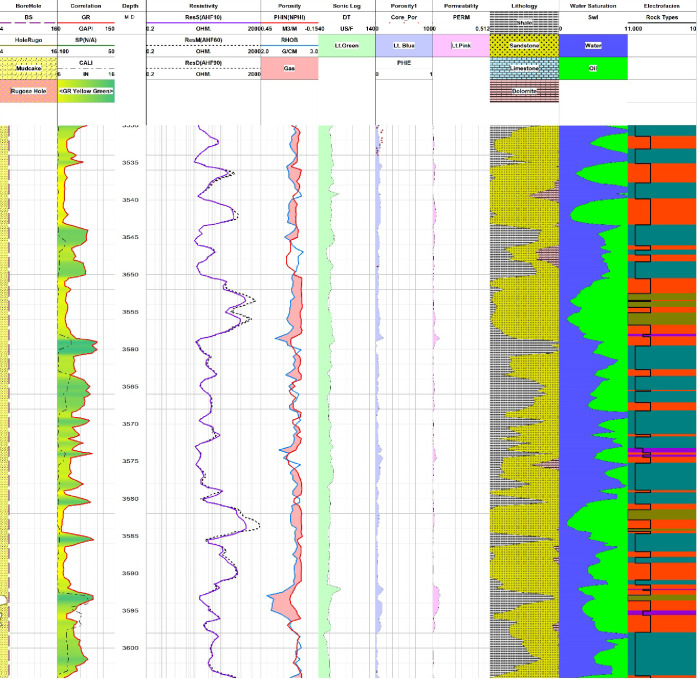
Pab Formation section in Zamzama-02 well showing results of petrophysical analysis along with rock type in the electrofacies track depicting rock reservoir quality. Reservoir quality displayed by curve named as Rock Types in the electrofacies track along with different colors illustrating individual rock types as shown in Table 3.

**Figure 7:**
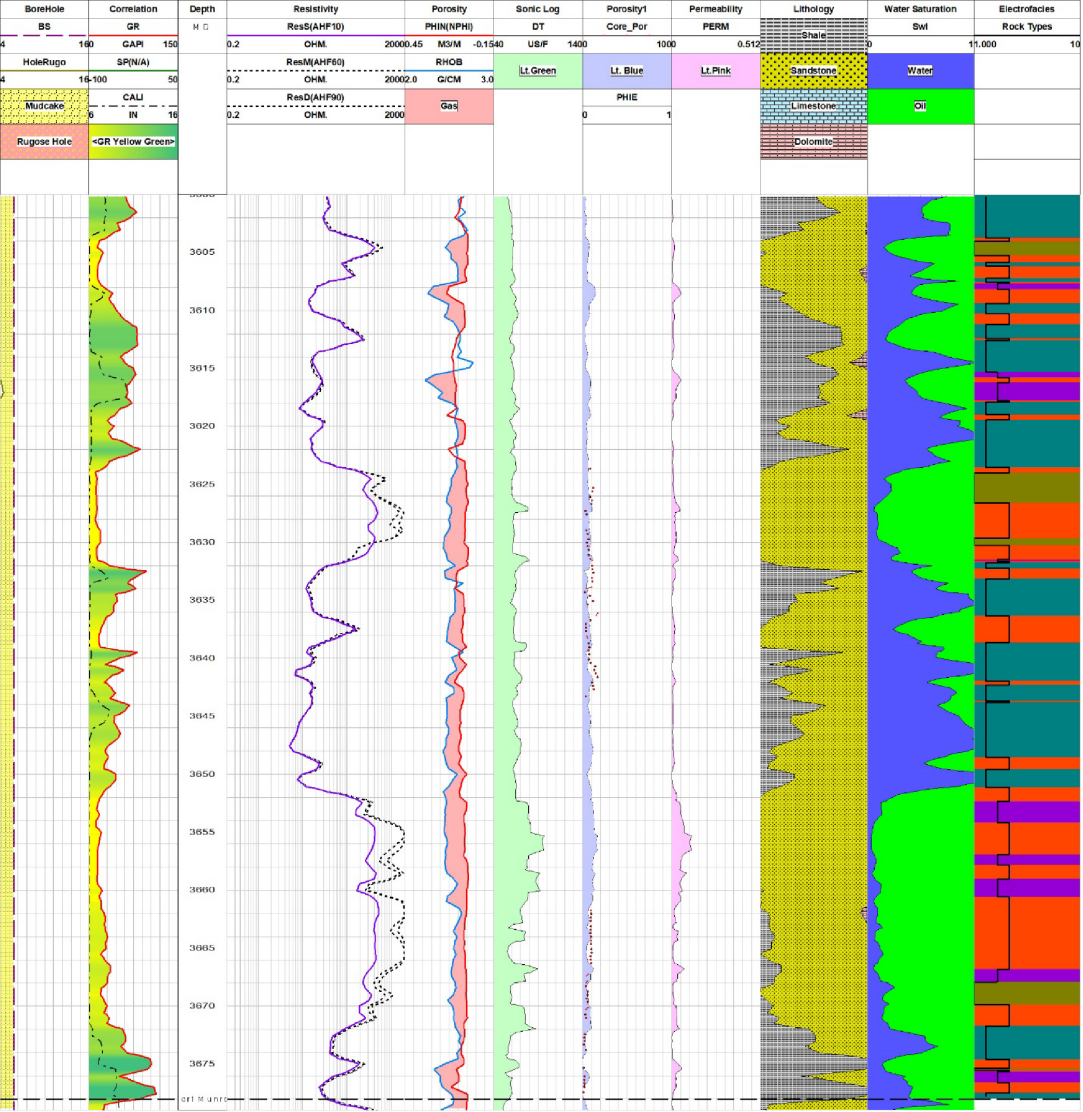
Pab Formation section in Zamzama-02 well showing results of petrophysical analysis along with rock type in the electrofacies track depicting rock reservoir quality. Reservoir quality is displayed by a curve named as Rock Types in the electrofacies track along with different colors illustrating individual rock types as shown in Table 3.

A pair plot shows the final graphical representation of cluster analysis for well Zamzama-02 (Figure 4). Figures 5–7 illustrate the results of petrophysical cluster analysis, where portions labeled with electrofacies in the last track demonstrate to which rock group the Pab Formation belongs to. The parameters used for cluster analysis include raw log for curves sonic and bulk density, whereas calculated core calibrated porosity, permeability, and water saturation represent the physical properties of pore fluids, shale, and mineral components as well as the textural properties of rocks.^18,37^ Core-calibrated porosity provides more accurate results for different reservoir parameters compared to porosities derived from conventional petrophysics.^15^ On the K-mean values of petrophysical properties tabulated in Table 2, the cluster analysis technique showed the quality of four rock types. According to K-mean values for every cluster, four rock types are devised to show the quality of the reservoir and are given in Table 3.

**Table 3:**
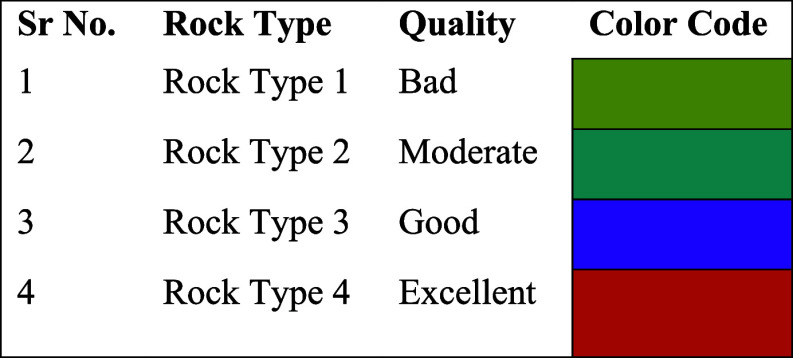
Reservoir Quality on the Basis of Rock Type along with the Respective Color Code

Using core-calibrated reservoir parameters as input curves helped with a more accurate rock type identification. The classification of reservoir quality is based on the responses of input parameters, which are further categorized into four rock types on the basis of cluster analysis. The hydrocarbon bearing zone is clearly correlated with excellent quality, which is rock type 4, marked by orange color. It represents purely sandstone lithology, which is a highly porous and permeable zone with good crossover between neutron and density porosity and with good hydrocarbon saturation results. Rock types 1 and 2 show bad to moderate quality due to a larger shale volume and higher irreducible water saturation. By comparing the results of cluster analysis to those of the lithology description, which is lithology derived from 3 and 4 mineral user-defined equations, rock types 1 and 2 are identified as shale sandstone with some intervals of dolomite. On the contrary, rock type 4 is mainly composed of sandstone with good reservoir storage capacity as it is evident from depths of 3472–3482 m, 3485–3493 m, 3502–3508 m, 3625–3630 m, and 3660–3665 m (Figures 5–7). Some intervals of the Pab Formation in Zamzama-02 well fall into the category of moderate quality, which is rock type 2. The zones of significant thickness for this rock type are 3515–3520 m, 3560–3580 m, and 3640–3650 m (Figures 5–7). Moreover, the accuracy of the identified rock types in the Pab Formation can be verified from the core results. When reservoir rock quality ranges from good to excellent, core-derived porosity and permeability results are good, as displayed in their respective tracks.

Sandstones of the Pab Formation are described as alluvial to coastal plain to lower shoreface mineralogically mature quartz arenites with minor amounts of feldspar. Grain size varies from silty to very coarse and pebbly.^38^ Furthermore, rock type identification based on cluster analysis was perfectly done despite differences in rock properties at different depths of the Pab Formation due to variations in rates of compaction and diagenetic processes.^39^

### 3.2 Well-based Facies Correlation

A well-to-well cross section between Zamzama North-1, 02, 05, and 03 has been created for stratigraphic correlation after the application of K-means clustering on each individual well. The cross section trended from north to south; Zamzama North-1 is the most northerly well within the Zamzama Gas Field. The template applied on the cross section contains raw curves like GR, ResD, and RHOB along with calculated curves and electrostatic curves displayed in their respective tracks. The Pab section is 212 m thick in this well, and the structural interpretation displays undisturbed sequence down to the Late Triassic by normal faulting or extensional tectonics.^40^ Overall, the Pab sequence in Zamzama North-1 is more shale-prone than in the majority of the wells to the south, as shown in the cross section. It consists of a highly interbedded series of sandstones and shales, as shown by the gamma ray responses, which display a high level of heterogeneity. In Zamzama-02, the Pab Formation is interpreted as 223.5 m thick. The contact with the Khadro Formation is based on a major shale break.^41,42^ However, it is dominated by pure sand in the lower part of the well. The Pab Formation is 230 m thick (measured depth) in Zamzama-05. The upper part of the formation is dominated by shaly sand, while clean sand is dominant in the lower part. The Pab Formation is 220.6 m thick (measured depth) in Zamzama-03. The top of the Pab Sandstone reservoir is distinguished by a very abrupt transition in lithology from sandstone to shales.^44^ The cross section indicates that reservoir quality is outstanding at the lower area of Zamzama-02 and 03, as indicated by rock type 4.

**Figure 8:**
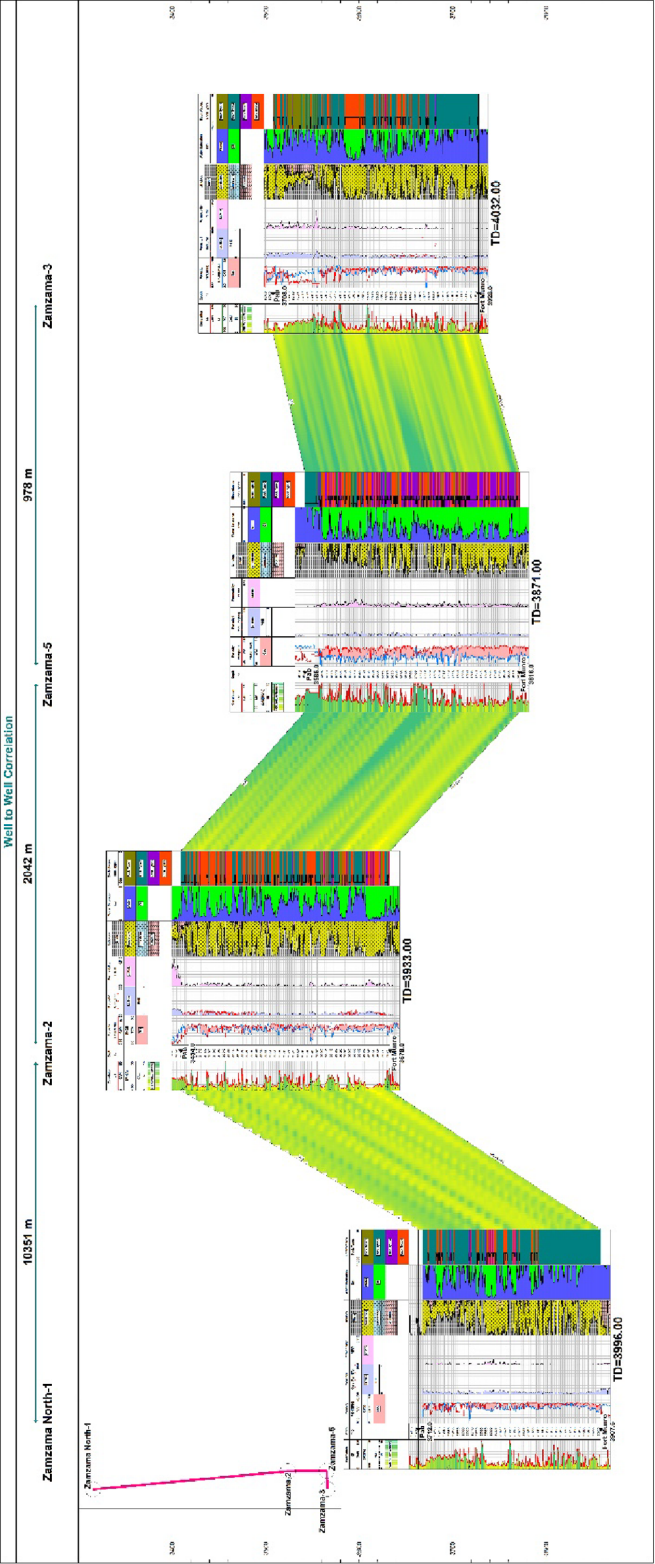
Well to Well correlation of Zamzama North-01, Zamzama-02, Zamzama-05, and Zamzama-03 well from north to southwest with reservoir parameters template.

A well-based facies correlation approach is employed for determining the distribution of facies in a reservoir across a particular line of section. Figure 8 represents a well-to-well cross section commencing from well Zamzama-North 01 and culminating at the Zamzama-03 well, passing through the two wells Zamzama-02 and Zamzama-05. On the basis of gamma-ray responses, these correlations were made for four wells with templates of reservoir parameters and rock type. The degree of correlativity between the Pab Formation and different wells can be seen through these cross sections.

The Zamzama-05 well, which is present in the south, has good to excellent reservoir quality as depicted by rock types 3 and 4 (Figure 6). The index map in the top left of the cross section shows the orientation of wells in Zamzama Gas Field. From Figure 6, it can be inferred that structurally, the Pab Formation is deeper in the northern part (in Zamzama North-01well), and in the central portion of the Zamzama Gas Field, it is found to be shallower (in Zamzama-02 well). Pab Formation depth is adequate at (Zamazama-05 and 03). The subsurface variations in depth could be interpreted as large north–south orientated and eastward verging thrusted anticline.^43^ The thickness of the Pab Formation is found to be approximately uniform across the Zamzama structure.

The limitation of the clustering technique is that different methods usually give different results. The variation in results is because of the different criteria for merging clusters. In the current study for K-means clustering, those input parameters are used for rock type identification, which is conventionally used for reservoir characterization. These input parameters are core calibrated, and the rock types are validated by core porosity and permeability findings.

## 4 Conclusions

The study aims to classify the Pab reservoir into different rock types based on the degree of variation and similarity among the clusters using logging records divided into equivalent intervals. Four rock types have been identified: rock type 1 and rock type 2 show bad and moderate rock type qualities and are identified as shaly sandstone with some intervals of dolomite. The majority of the Pab Formation in the Zamzama-02 well is classified as excellent quality with rock type 4 being the most common. Rock type 4, which is of excellent quality, is mainly composed of sandstone with a good reservoir storage capacity. The cross section indicates that reservoir quality is excellent at the lower parts of Zamzama-02 and 05, as indicated by rock type 4. Zamzama North-01 has moderate reservoir quality, as indicated by rock type 2. On the other hand, the Zamzama-03 well, which is present in the southwest, has good to moderate reservoir quality, as depicted by rock types 3 and 2, respectively. The electrochemical properties were continuously predicted along the drill core in the studied wells. The use of this methodology in defining rock type is cost-effective, less time-consuming in the demarcation of zones of interest, and more accurate than manual petrophysical analysis of the heterogeneous and thick Pab Formation. The research approach could be applied not only to the exploitation of the heterogeneous Pab Formation but also to other heterogeneous sandstone reservoirs.

## Data Availability

The authors do not have permission to share data.
